# Transcriptome analysis and transient transformation suggest an ancient duplicated MYB transcription factor as a candidate gene for leaf red coloration in peach

**DOI:** 10.1186/s12870-014-0388-y

**Published:** 2014-12-31

**Authors:** Ying Zhou, Hui Zhou, Kui Lin-Wang, Sornkanok Vimolmangkang, Richard V Espley, Lu Wang, Andrew C Allan, Yuepeng Han

**Affiliations:** Key Laboratory of Plant Germplasm Enhancement and Specialty Agriculture, Wuhan Botanical Garden of the Chinese Academy of Sciences, 430074 Wuhan, People’s Republic of China; Graduate University of Chinese Academy of Sciences, 19A Yuquanlu, Beijing, 100049 People’s Republic of China; The New Zealand Institute for Plant & Food Research Ltd, (Plant and Food Research), Mt Albert Research Centre, Private Bag 92169, Auckland, New Zealand; Department of Pharmacognosy and Pharmaceutical Botany, Faculty of Pharmaceutical Sciences, Chulalongkorn University, 10330 Bangkok, Thailand; School of Biological Sciences, University of Auckland, Private Bag 92019, Auckland, New Zealand

**Keywords:** *Prunus persica*, Anthocyanin coloration, Gene duplication, Transcriptome analysis

## Abstract

**Background:**

Leaf red coloration is an important characteristic in many plant species, including cultivars of ornamental peach (*Prunus persica*). Peach leaf color is controlled by a single *Gr* gene on linkage group 6, with a red allele dominant over the green allele. Here, we report the identification of a candidate gene of *Gr* in peach.

**Results:**

The red coloration of peach leaves is due to accumulation of anthocyanin pigments, which is regulated at the transcriptional level. Based on transcriptome comparison between red- and green-colored leaves, an MYB transcription regulator *PpMYB10.4* in the *Gr* interval was identified to regulate anthocyanin pigmentation in peach leaf. Transient expression of *PpMYB10.4* in tobacco and peach leaves can induce anthocyain accumulation. Moreover, a functional *MYB* gene *PpMYB10.2* on linkage group 3, which is homologous to *PpMYB10.4*, is also expressed in both red- and green-colored leaves, but plays no role in leaf red coloration. This suggests a complex mechanism underlying anthocyanin accumulation in peach leaf. In addition, *PpMYB10.4* and other anthocyanin-activating *MYB* genes in Rosaceae responsible for anthocyanin accumulation in fruit are dated to a common ancestor about 70 million years ago (MYA). However, *PpMYB10.4* has diverged from these anthocyanin-activating *MYBs* to generate a new gene family, which regulates anthocyanin accumulation in vegetative organs such as leaves.

**Conclusions:**

Activation of an ancient duplicated *MYB* gene *PpMYB10.4* in the *Gr* interval on LG 6, which represents a novel branch of anthocyanin-activating *MYB* genes in Rosaceae, is able to activate leaf red coloration in peach.

**Electronic supplementary material:**

The online version of this article (doi:10.1186/s12870-014-0388-y) contains supplementary material, which is available to authorized users.

## Background

Peach [*Prunus persica* L. (Batsch)], a member of the Rosaceae family, is an important fruit tree crop worldwide. It is a diploid with a small genome size of ~ 230 Mb [1]. Besides providing delicious fruit, peach trees are extensively used in ornamental plantings. In China, ornamental peach has been cultivated for landscape or patio plants for thousands of years. The color of flowers and leaves is one of the most attractive characteristics, which contribute to the ornamental value of plants [2]. In peach, red color is caused mainly by the accumulation of anthocyanins.

Anthocyanins are the largest group of water-soluble pigments in the plant kingdom and belong to the family of compounds known as flavonoids. Anthocyanins are stored in the central vacuole and responsible for the red, blue and purple colors in a wide range of plant tissues, including stems, leaves, roots, flowers, fruits and seeds [3,4]. Anthocyanins are synthesized via flavonoid biosynthetic pathway and display a wide range of biological functions such as attracting pollinators and seed dispersers and protecting plants against attack by pathogenic organisms and UV radiation [5]. In addition, anthocyanins have a beneficial role in human health because they exhibit a wide range of biological activities such as antioxidant, anti-inflammatory, antimicrobial and anti-cancer activities [6]. Therefore, anthocyanins have long been the subject of investigation by botanists and plant physiologists.

The conserved biosynthetic pathway of anthocyanins has been well established in ornamental plants such as petunia and snapdragon [7,8]. The biosynthesis of anthocyanins begins with condensation of coumaroyl-CoA with malonyl-CoA to form naringenin chalcone by chalcone synthase (CHS). The chalcone is converted to naringenin by chalcone isomerase (CHI). Flavanone 3-hydroxylase (F3H) then catalyzes hydroxylation of naringenin to yield dihydrokaempferol (DHK). DHK can be further hydroxylated to produce dihydromyricetin (DHM) or dihydroquercetin (DHQ) by flavonoid 3′, 5′-hydroxylase (F3′5′H) or flavonoid 3′-hydroxylase (F3′H), respectively. DHK, DHM and DHQ are converted into anthocyanidins by dihydroflavonol reductase (DFR) and leucoanthocyanidin dioxygenase (LODX). Finally, anthocyanidin is glycosylated by UDP glucose: flavonoid 3-*O*-glucosyltransferase (UFGT) to generate anthocyanin. To date, anthocyanin pathway genes have been isolated and characterized in a variety of model plants such as petunia, snapdragon, and *Arabidopsis* [4].

The anthocyanin pathway genes are regulated at the transcriptional level by three types of regulatory genes encoding R2R3 MYB, basic helix-loop-helix (bHLH) and WD40 proteins, respectively [5]. These regulators interact with each other to form a MBW complex that binds to promoters and induces transcription of genes of the anthocyanin biosynthetic pathway. To date, molecular mechanisms underlying anthocyanin biosynthesis in fruits has been widely reported. For example, in grape two adjacent MYB transcription factors (TFs) *VvMYBA1* and *VvMYBA2* are responsible for the activation of *UFGT* gene, thus, have a regulatory effect on anthocyanin accumulation [9]. Similarly, three transcription factors which appear to be allelic, *MdMYB10*, *MdMYB1*, and *MdMYBA,* have been isolated and characterized in apple [10-12]. In other Rosaceous fruits, such as pear, raspberry, strawberry and plum, homologues of *MYB10* have been isolated [13]. More recently, a MYB gene, designated *Ruby*, has been identified in citrus and its activation is responsible for the accumulation of anthocyanins in blood oranges [14]. Besides fruit, anthocyanin accumulation in foliage is also a wide-spread phenomenon and the role of anthocyanins in senescing leaves has been investigated in temperate deciduous plants [15]. However, there are few reports on the molecular mechanism underlying red coloration in ornamental trees or other deciduous trees.

Peach leaf color is controlled by a single gene (*Gr*), with red allele dominant over green allele [16]. Recently, the *Gr* locus has been mapped to the middle region of linkage group (LG) 6 [17]. In peach, two MYB TFs have been reported to control anthocyanin coloration in fruit skin [18] and flower [19]. Recently, a cluster of three *MYBs*, termed *MYB10.1*, *MYB10.2* and *MYB10.3*, on the same genomic fragment where the Anther color (*Ag*) trait is located on linkage group 3, were implicated in regulating fruit anthocyanin biosynthesis [20]. Here, we report the identification of a MYB TF in the *Gr* interval, which functions as a candidate *Gr* gene for leaf red coloration of ‘Hongyetao’, a popular ornamental peach cultivar in China. The distinctive features of this cultivar are its attractive red leaf coloration and pink-red flowers. The functionality of the peach *MYB* gene has been demonstrated via transient expression in both tobacco and peach. Our results add to the comprehensive understanding of the mechanisms underlying anthocyanin biosynthesis in peach.

## Results

### Anthocyanin accumulation in different colored tissues of peach

Anthocyanin contents were measured in different tissues of two cultivars Hongyetao (HYT) and Mantianhong (MTH) (Table 1). ‘HYT’ is an ornamental cultivar. It produces small brown-skinned fruits with white flesh and has purple-red leaves, red stems and pink-red flowers in the spring. However, the color of the leaves fades to green with maturity. The pink-red flower contains the highest level of anthocyanins, followed by the red leaf and stem, while the mature green-colored leaf and fruit accumulate little anthocyanin. ‘MTH’ has green leaves, red flowers, and white-fleshed fruits. The red flower contains high level of anthocyanins, while the anthocyanin content is very low in other tissues, including leaf, stem and fruit. In summary, anthocyanin accumulation in red-colored tissues is significantly higher than in non-red tissues, which is similar to previous reports that anthocyanin accumulation is responsible for red coloration in peach [18-23].

**Table 1:** **Anthocyanin contents in different tissues of peach cv. Hongyetao and Mantianhong**

**Tissue**	**Cultivar**	**Color**	**Anthocyanin content (mg/100 g FW)**
Young leaf in spring	Hongyetao	Red	49.52 ± 1.43
	Mantianhong	Green	1.62 ± 0.53
Mature leaf in spring	Hongyetao	Green	1.13 ± 0.72
	Mantianhong	Green	1.53 ± 0.14
1-year-old stem in spring	Hongyetao	Red	21.77 ± 3.13
	Mantianhong	Green	3.02 ± 0.09
Flower at full-bloom stage	Hongyetao	Red	79.13 ± 2.94
	Mantianhong	Red	71.22 ± 2.71
Flesh at ripening stage	Hongyetao	White	1.96 ± 0.62
	Mantianhong	White	1.45 ± 0.50

### Identification of candidate gene in the peach *Gr* interval by comparative transcriptome analysis

The *Gr* interval has been mapped to an interval flanked by two SSR markers BPPCT009 and CPDCT041 on LG6 [17]. Comparison of primer sequences of the two SSR markers against the peach draft genome revealed that the *Gr* interval is about 7.9 Mb in physical size, ranging from 11.9 Mb to 19.8 Mb on LG6. To identify the candidate *Gr* gene, transcriptomes of young leaves from cv. HYT and MTH were sequenced using Illumina RNA-seq technology, yielding 16 and 11 million transcript reads, respectively. These reads were mapped onto the peach reference genome and the mapping result was deposited in NCBI SRA database with accession nos. SRX767357 and SRX796311. Gene expression level was estimated using FPKM (fragments per kilobase of exon per million fragments mapped) value and a threshold of 1.5 times fold-change was used to separate the genes differentially and non-differentially expressed. Of the 129 genes in the *Gr* interval, 18 genes were identified to be differentially expressed between red- and green-colored leaves (Table 2). Of these genes, only one (ppa018744m) encoding a transcription factor homologous to *Arabidopsis AtMYB113* is related to anthocyanin biosynthesis. The gene, designated *PpMYB10.4*, showed 239.5 times higher level of expression in red-colored leaves than in green-colored leaves. Besides the *PpMYB10.4* gene, another *AtMYB113* homologue outside the *Gr* interval on LG3, termed *PpMYB10.2* [20], was identified in the peach leaf transcriptome. However, its expression level was 0.3-fold lower in red-colored leaves than in green-colored leaves.

**Table 2:** **Genes located in the** ***Gr*** **interval and differentially expressed between red- and green-young leaves of peach cv. HYT and MTH, respectively**

Gene ID*	**FPKM**			**Transcript**		**Description**
	**HYT**	**MTH**	**HYT/MTH**	**Start**	**Stop**	
ppa002898m	28.22	41.12	0.69	11968323	11970611	Unknown protein
ppa019862m	14.53	22.03	0.66	13127613	13129381	Heat shock family protein
ppa007092m	0.27	0.14	1.94	13271592	13273679	GDSL-motif lipase/hydrolase family protein
ppa023596m	0.71	1.44	0.49	14747032	14750178	Disease resistance protein (TIR-NBS-LRR class)
ppa017706m	0.05	0.00	0.05/0	15538309	15540816	Glycosyl hydrolase family 17 protein
ppa015480m	8.24	21.17	0.39	15635236	15637392	WRKY51; transcription factor
ppa011208m	8.07	14.83	0.54	15680828	15682231	Unknown protein
ppa008614m	9.94	15.64	0.64	15941096	15944967	Unknown protein
**ppa018744m**	**17.66**	**0.07**	**239.50**	**16147279**	**16150033**	**MYB113 (myb domain protein 113)**
ppa012771m	11.93	21.16	0.56	16601995	16602908	Heavy-metal-associated domain-containing protein
ppa020536m	2.36	1.13	2.10	16942666	16944399	Unknown protein
ppa011539m	6.77	4.41	1.53	17711411	17714182	*Arabidopsis* Rab GTPase homolog H1e
ppa017055m	4.65	17.68	0.26	17918320	17920029	UDP-glucosyl transferase 85A2
ppa004086m	9.39	5.54	1.69	18525587	18531367	PHOSPHOFRUCTOKINASE 5
ppa017857m	2.44	0.94	2.58	18746742	18750038	RPM1 (resistance to *P. syringae* pv maculicola 1)
ppa026318m	0.58	6.01	0.10	18822274	18825206	RPM1 (resistance to *P. syringae* pv maculicola 1)
ppa005809m	0.44	0.09	5.12	19048758	19053125	RAB GDP-dissociation inhibitor
ppa025098m	0.55	3.82	0.15	19150519	19153384	Polygalacturonase

*The candidate gene in the *Gr* locus is indicated in bold.

Subsequently, we checked the expression levels of anthocyanin structural genes and found *PpCHS*, *PpCHI*, *PpF3H*, *PpF3′H*, *PpDFR*, and *PpLDOX* showed 1.5-, 1.6-, 2.1-, 2.7-, 4.5-, and 4.9-fold higher levels of expression, respectively, in red-colored leaves than in green-colored leaves. *PpUFGT* was highly expressed in red leaves, whereas, its transcript was almost undetectable in green leaves. This demonstrates that accumulation of anthocyanin in peach leaf is regulated at the transcriptional level. Since anthocyanin biosynthesis is regulated by the MBW complex [5], we also investigated anthocyanin-related bHLH and WD40 TFs in the peach leaf transcriptome. Two homologues of *AtGL3* (*PpbHLH3* and *PpbHLH33*) and two homologues of *AtTTG1* (*PpWD40A1* and *PpWD40A2*) were identified. *PpbHLH3* and *PpbHLH33* had 0.6- and 0.1-fold higher levels of expression level, respectively, in red leaves than in green leaves. In contrast, *PpWD40A1* and *PpWD40A2* had 0.3 and 0.1-fold lower levels of expression, respectively, in red leaves than in green leaves. Taken together, all the results suggest that *PpMYB10.4* is the candidate gene for the *Gr* locus.

### Two clusters of MYB-type anthocyanin regulators in the peach genome

**Figure 1:**
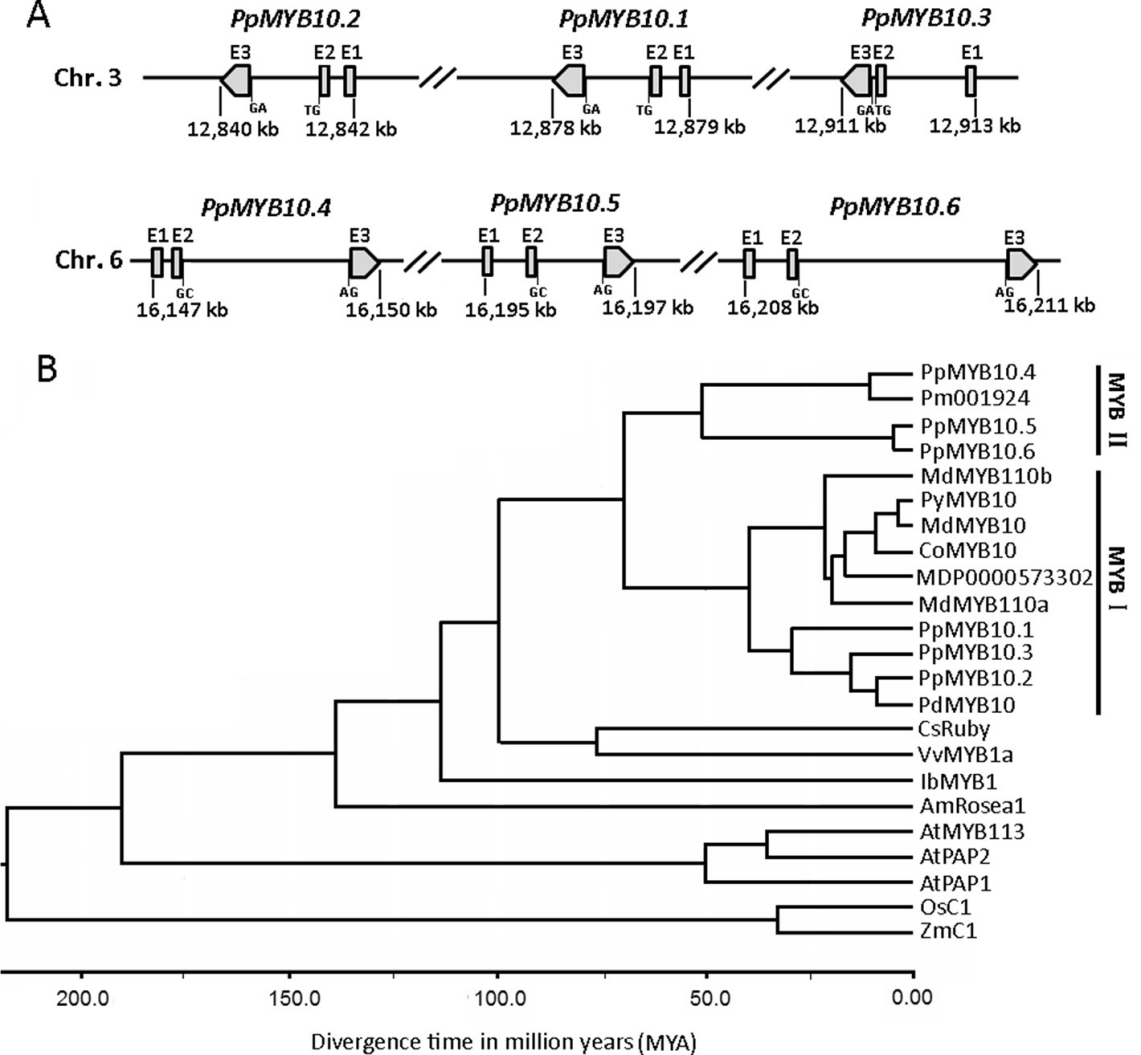
**Six anthocyanin-related MYB genes in the peach genome. A**, Structural feature and chromosomal position of the six peach MYB genes. **B**, Estimated divergence time between anthocyanin-related MYB genes in plants based on aligned nucleotide sequences using Bayesian MCMC analysis. The GenBank accession numbers are as follows: *Prunus domestica* PdMYB10 (ABX71492); *Malus* × *domestica* MdMYB10 (AFC88038), MdMYB110a (JN711473), and MdMYB110b (JN711474); *Pyrus communis* PyMYB10 (JX403957); *Cydonia oblonga* CoMYB10 (EU153571); *Citrus sinensis* CsRuby (AFB73909); *Vitis vinifera* VvMYB1a (ABB87014); *Ipomoea batatas* IbMYB1 (BAF45114); *Arabidopsis* AtPAP1 (NP_176057), AtPAP2 (NP_176813), AtMYB11 (NP_191820), and AtMYB113 (NP_176811); *Antirrhinum majus* AmRosea1 (ABB83826), *Zea mays* ZmC1 (NM_001112540); and *Oryza sativa* OsC1 (HQ379703). Pm001924 and MDP0000573302 are extracted from the released genome sequences of *Prunus mume* [27] and apple, respectively.

To determinate whether multiple MYB genes are involved in the regulation of anthocyanin accumulation in peach leaves, we compared the cDNA sequences of *PpMYB10.2* and *PpMYB10.4* against the draft genome of peach cv. Lovell using blastn [1]. As a result, *PpMYB10.2* and its two paralogs, termed *PpMYB10.1* and *PpMYB10.3* [20], were located next to each other within a 72 kb region on chromosome 3, while *PpMYB10.4* and its two paralogs (*PpMYB10.5* and *PpMYB10.6*) were clustered within a 63 kb region on chromosome 6 (Figure 1A). Accession numbers of *PpMYB10.1* to *PpMYB10.6* at the Genome Database for Rosaceae (GDR, http://www.rosaceae.org/) were listed in Additional file 1: Table S1. *PpMYB10.2* was identical in sequence to *PpMYB10* previously isolated from peach fruit [13]. *PpMYB10.3* and *PpMYB10.1* have recently been implicated in peach fruit pigmentation [20]. All the six MYB TFs consist of three exons separated by two introns. The consensus sequences, GC and AG, were found at the 5′ and 3′-borders of the two introns of *PpMYB10.1* to *PpMYB10.3*, strictly following the “GT–AG” splicing site of the eukaryotic introns proposed by Breathnach and Chambon [24]. In contrast, “GT–AG” and “GC-AG” splicing sites were observed for the first and second introns of *PpMYB10.4* to *PpMYB10.6*, respectively.

The evolutionary history assay revealed that the ancestral *MYB* gene at early stages of Rosaceae have undergone a duplication, ~ 70 million years ago (MYA), to generate the two gene families, designated MYBIand MYBII (Figure 1B). MYBI consists of *PpMYB10.1* to *PpMYB10.3* and their homologues such as *MdMYB10* [12], *MdMYB110a* [25] in *Malus* and *PyMYB10* [26] in *Pyrus* and *PdMYB10* in *Prunus domestica*, while MYBII contains *PpMYB10.4* to *PpMYB10.6* and their homologues such as *PmMYB* gene in *Prunus mume* [27].

### Expression profiling of anthocyanin related genes in red- and green peach leaves by qRT-PCR

**Figure 2:**
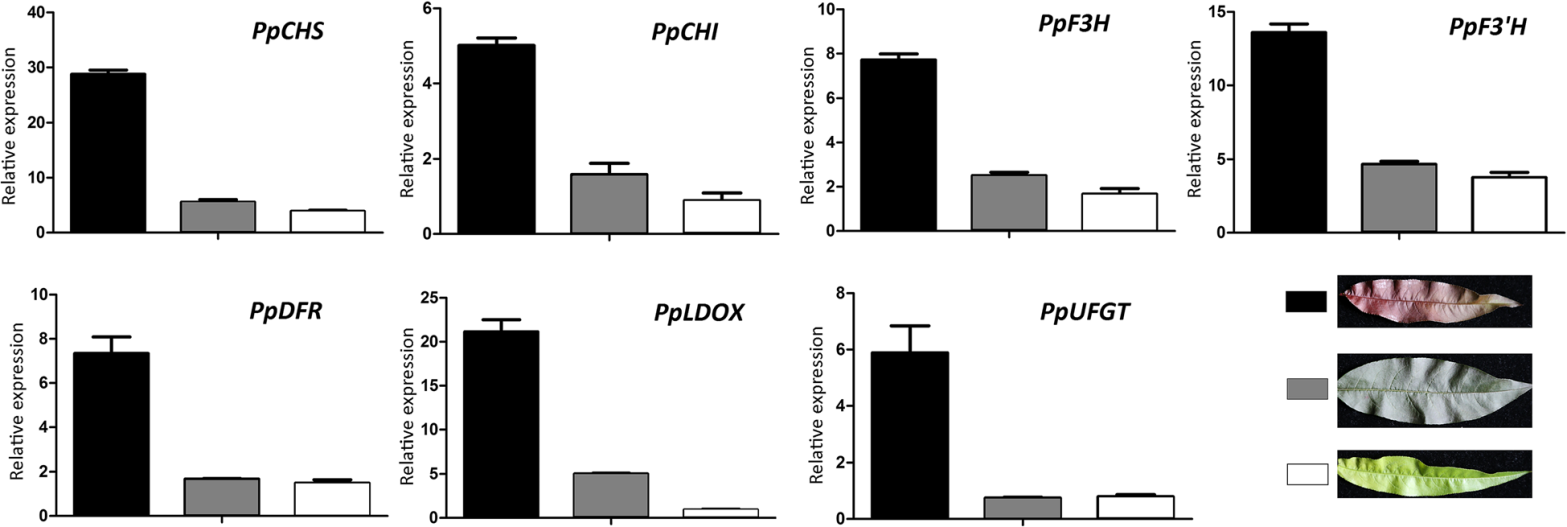
**Expression levels of anthocyanin pathway genes in red- or green-colored leaves of different cultivars grown in spring season.** The black, grey, and white boxes represent young leaf of cv. HYT, mature leaf of cv. HYT, and young leaf of cv. MTH, respectively.

**Figure 3:**
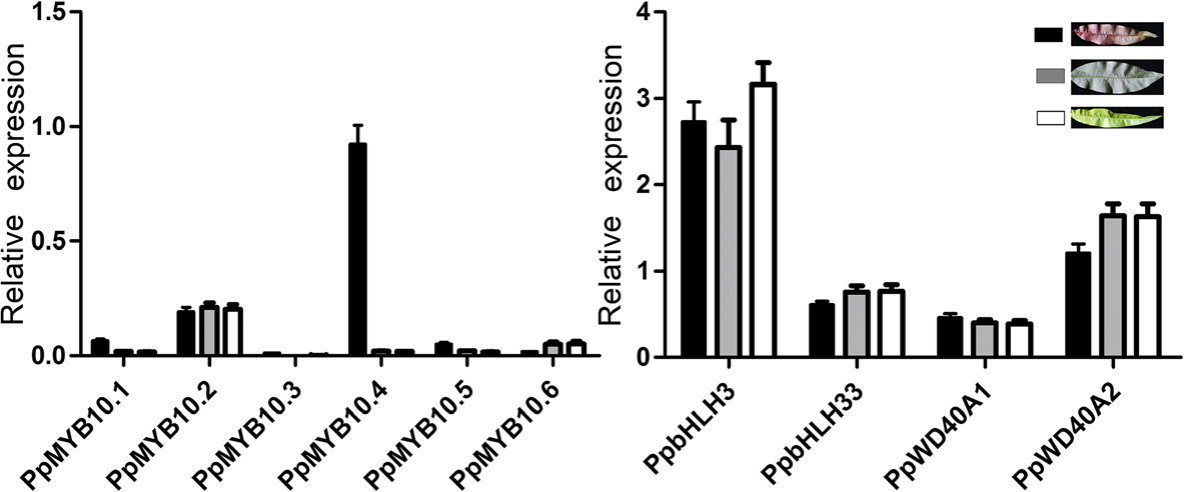
**Expression profiles of anthocyanin regulatory genes in spring season leaves of two peach cultivars.** The black, grey, and white boxes represent red-colored young leaves of cv. HYT, green-colored mature leaves of cv. HYT, and green-colored young leaves of cv. MTH, respectively.

To validate the RNA-seq-based gene expression profiles, the expression level of anthocyanin biosynthesis genes was examined in leaves of cv. HYT and MTH using qRT-PCR. All the biosynthetic pathway genes, including *CHI*, *CHS*, *DFR*, *F3′H*, *F3H*, *LDOX*, and *UFGT*, showed significantly higher level of expression in red leaves than in green leaves (Figure 2). For regulator genes, the *PpbHLH* and *PpWD40* genes were expressed in leaves, but showed no difference in expression level between red- and green variants (Figure 3). Of the six *MYB* genes, four (i.e. *PpMYB10.1*, *PpMYB10.3*, *PpMYB10.5*, and *PpMYB10.6*) showed extremely low expression in both red- and green- leaves. *PpMYB10.2* gene was expressed in leaves, but showed no difference in expression level between red- and green-colored leaves. In contrast, the expression level of *PpMYB10.4* gene in red- leaves was significantly higher than those in green- leaves.

**Figure 4:**
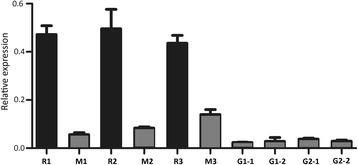
**Expression levels of** ***PpMYB10*** **.** ***4*** **gene in different colored leaves of peach.** R1, young leaves of cv. HYT in spring; M1, mature leaves of cv. HYT in late spring; R2, young leaves of cv. HYT in summer; M2, mature leaves of cv. HYT in summer; R3, young leaves of cv. HYT in autumn; M3, mature leaves of cv. HYT in autumn; G1-1, young leaves of cv. MTH in spring, G1-2, mature leaves of cv. MTH in spring; G2-1, young leaves of cv. Baihuabitao in spring; G2-2, mature leaves of cv. Baihuabitao in spring. The black and grey boxes indicate red- and green-colored leaves, respectively.

In addition, the expression profile of *PpMYB10.4* gene was also examined in leaves at different developmental stages and a second green foliage cultivar ‘Baihuabitao’ was included in the qRT-PCR analysis (Figure 4). The expression levels of *PpMYB10.4* gene were significantly higher in young leaves of cv. Hongyetao than those in mature leaves in all three seasons, including spring, summer and autumn. However, the expression levels of *PpMYB10.4* gene were very low in both young and mature leaves of cvs. Baihuabitao and Mantianhong. The result of qRT-PCR is consistent with that of RNA-seq-based gene expression profiling, which confirms that activation of *PpMYB10.4* gene in red leaves.

### *PpMYB10.4* is a functional regulator that induces anthocyanin accumulation in tobacco and peach

**Figure 5:**
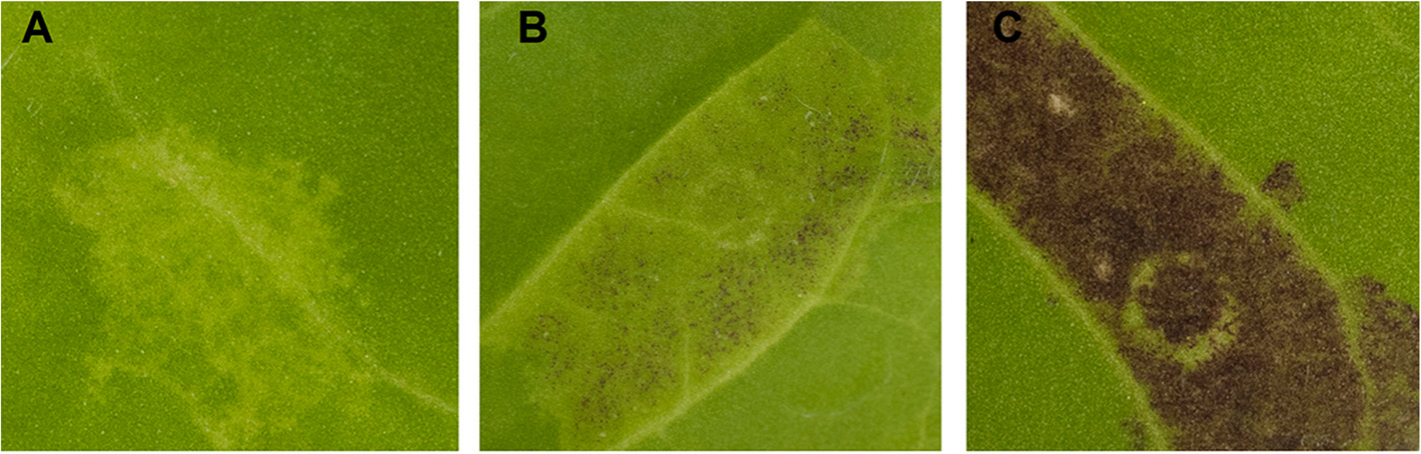
**Transient expression of peach** ***PpMYB10*** **.** ***4*** **gene in tobacco leaf. A**, **B**, and **C** indicate infiltration sites 7 days after transformation with *PpbHLH3*, *PpMYB10.4*, and *PpMYB10.4*/*PpbHLH3*, respectively.

Transcriptional activity of *PpMYB10.4* was initially tested using a tobacco transient colour assay. PpMYB10.4 and bHLH3 were syringe-infiltrated into the underside of expanding *Nicotiana tabacum* leaves. No pigmentation was observed at infiltration sites 7 days after transformation with *PpbHLH3* (Figure 5A), while a slight pigmentation was observed with infiltration of *PpMYB10.4* (Figure 5B). An intense pigmentation was detected at infiltration sites 7 days after transformation with both *PpMYB10.4* and *PpbHLH3* (Figure 5C).

**Figure 6:**
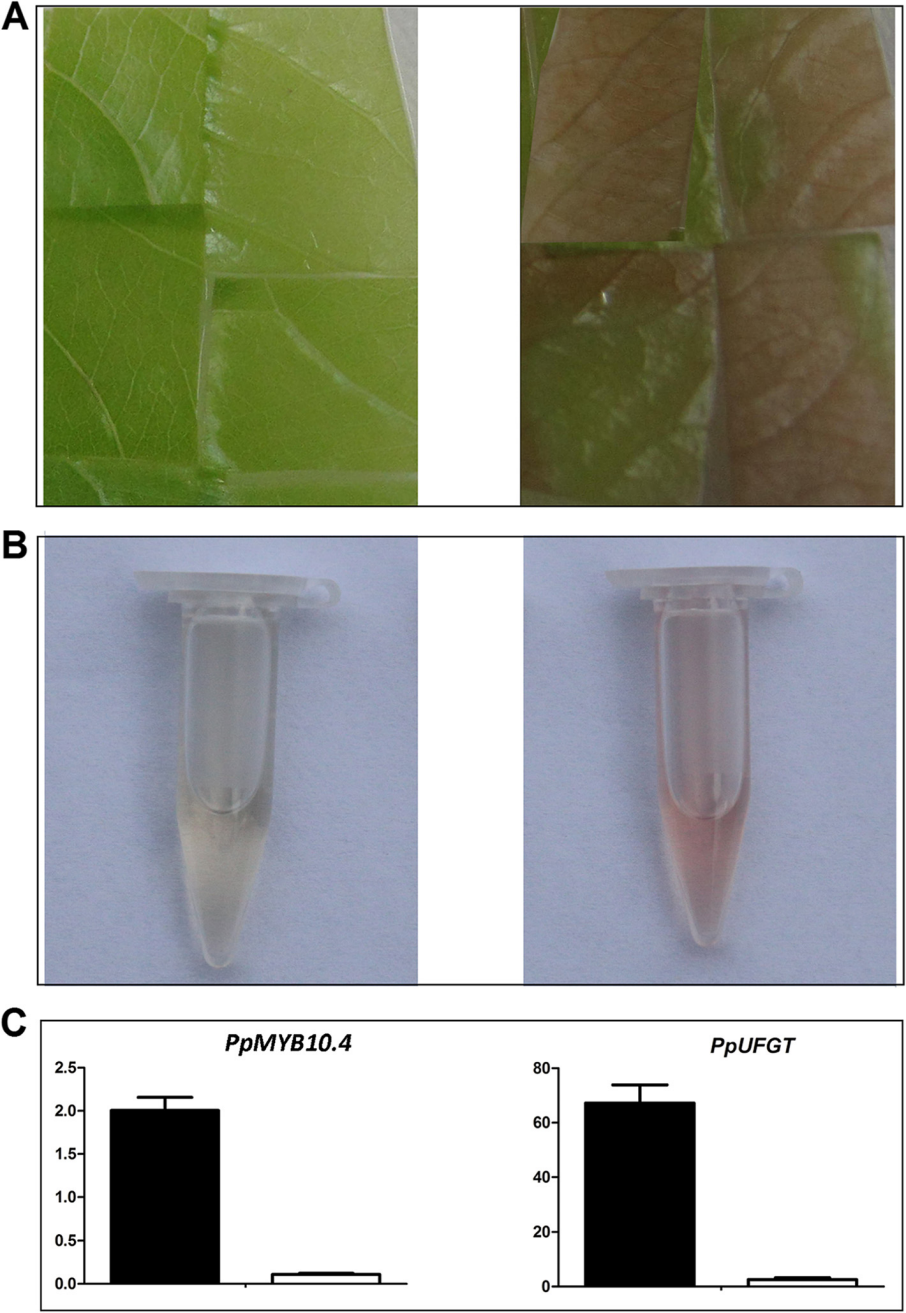
**Functional analysis of peach** ***PpMYB10*** **.** ***4*** **using transient expression assay. A**, Transient expression of *PpMYB10.4* gene (right) together with an empty vector as control (left) in young leaf of cv. Mantianhong. **B**, Extraction of anthocyanins. **C**, Expression levels of *PpMYB10.4* and *PpUFGT* in peach leaves transformed with *PpMYB10.4* (black box) and empty vector (white box), respectively.

The functionality of *PpMYB10.4* was further validated by particle bombardment-mediated transient expression in green-colored young leaves of cv. MTH. The leaves turned red 2 days after transformation with *PpMYB10.4*, but the leaves transformed with empty vector (EV) were still green in color (Figure 6A). Anthocyanin extraction results showed that the peach leaves transformed with *PpMYB10.4* contained anthocyanin, but not for the EV-transformed leaves (Figure 6B). Moreover, *PpMYB10.4* was highly expressed in leaves transformed with *PpMYB10.4*, while its transcript level was extremely low in leaves transformed with EV (Figure 6C). Likewise, *PpUFGT* showed over 30 times higher expression level in leaves transformed with *PpMYB10.4* than in leaves transformed with EV.

**Figure 7:**
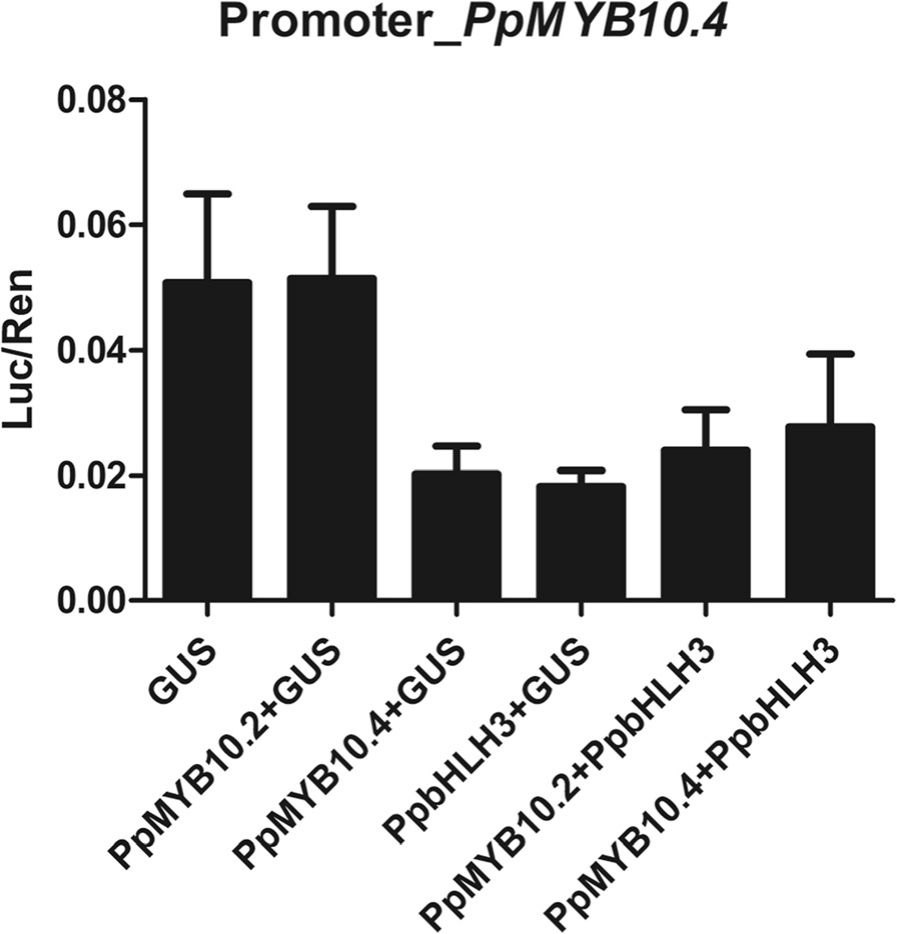
**Analysis of the effect of peach** ***MYB*** **genes on the activation of the promoter of** ***PpMYB10*** **.** ***4*** **in red foliage cv.** Hongyetao. Agrobacterium carrying a 35S:Gus plasmid is used as a negative control. Error bars are SE for 4 replicate reactions.

It has previously been shown the apple MYB10 can regulate is own expression [28]. A dual luciferase assay was conducted to clarify if the expression of *PpMYB10.4* is auto-regulated or can be regulated by, for example, PpMYB10.2. However, no interaction was detected between PpMYB10.2 and the promoter of *PpMYB10.4*, and PpMYB10.4 had no influence on its own expression in this transient assay (Figure 7).

### Sequence polymorphisms in the promoter region of *PpMYB10.4*

**Figure 8:**
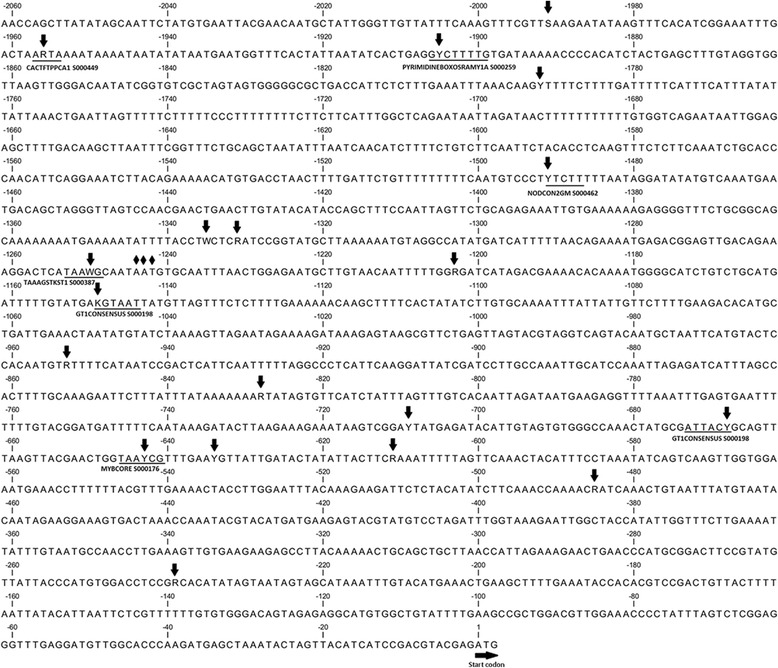
**Nucleotide sequence of the promoter region of** ***PpMYB10*** **.** ***4*** **.** The positions of SNPs and one 3-bp insertion-deletion are indicated with black arrows and diamond, respectively, while cis-regulatory motifs are highlighted with underlines.

While there are differences in the expression profile of *PpMYB10.4* between red- and green-colored leaves, the coding sequences of *PpMYB10.4* are identical between cv. HTY and MTH. Hence, a pair of primers 5′-GGATCTCGCCGCTGTTTCTG-3′ and 5′-TCTCACTCCCGAAGAACTATCCAT-3′ was designed to amplify the promoter genomic regions of *PpMYB10.4* in cv. HTY and MTH. The promoter sequences of *PpMYB10.4* from cvs. HTY, MTH, and Lovell were aligned and 18 single nucleotide polymorphisms (SNPs) and a 3-bp indel were identified within a 2.06-kb region upstream the *PpMYB10.4* start codon (Figure 8). Of these SNPs, seven were located within potential motifs, which were identified using the PLACE program [29]. Among these motifs, one MYBCORE is a potential binding site for MYB-type anthocyanin regulators. However, the T/G SNP in the MYBCORE site is found in the promoter of both ‘HYT’ and ‘MTH’, suggesting that it is not causative for the red leaf coloration. There was a 3-bp insertion found in the promoter of cv. HYT, and the 3-bp indel site was polymorphic. However, the 3-bp insertion was not found in the promoter of cv. MTH and Lovell. To test if the 3-bp indel is related to the red leaf coloration, a pair of primers flanking the 3-bp indel (5′-TTTTACCTTCTCGATCCGGTAT-3′ and 5′-AATTGTTACAAGCATTCTCCAGTT-3′) was then designed to amplify products in diverse peach cultivars, including ‘Datuanmilu’, ‘Gangshanbai’, ‘Huyou002’, ‘Jinyuan’, ‘May Fire’, ‘Nanfangzaohong’, ‘Ruiguangmeiyu’, ‘Wuyuexian’, ‘Xizhuangyihao’, and ‘Zhaoxia’. All these cultivars have green-colored leaves. However, the 3-bp insertion was also found in the promoter of four cultivars, Huyou002, Nanfangzaohong, Xizhuangyihao, and Zhaoxia. This suggests the indel is unlikely to be responsible for leaf red coloration in peach.

We also identified repetitive elements in the promoter sequences of *PpMYB10.4* using the program RepeatMasker (http://www.repeatmasker.org/). One transposon-like fragment 280 bp in size was found to be located 692 bp upstream of the ATG translation start codon. However, the transposon-like fragment is almost identical in nucleotide sequence between HTY and MTH. This suggests that this transposable element is unlikely to be responsible for activation of *PpMYB10.4*.

## Discussion

### The mechanism underlying anthocyanin accumulation in peach leaves

In many plant species, anthocyanin accumulation is controlled primarily via transcriptional regulation by R2R3 MYB transcription factors [4]. Here, an R2R3 anthocyanin-activating *MYB* gene *PpMYB10.4*, which located in the *Gr* interval, is shown to be the candidate *Gr* gene for red leaf coloration in peach. Moreover, our study reveals that the *PpMYB10.4* homologue *PpMYB10.2* is also expressed in peach leaf. Previous study has demonstrated that *PpMYB10.2* is a functional gene responsible for anthocyanin pigmentation in peach skin [18,20]. However, the *PpMYB10.2* expression alone is unlikely to induce anthocyanin pigmentation in the peach leaf. Firstly, PpMYB10.2 has no effect on the induction of the *PpMYB10.4* expression. Also, *PpMYB10.2* has similar or lower levels of expression in red leaves than green leaves. Transcriptome analysis revealed that two homologues (ppa019522m and ppa010846m) of *AtMYBL2*, a negative regulator of anthocyanin biosynthesis in *Arabidopsis*, are expressed in leaf. These *MYB* repressors may compete with MYB activators for binding sites of bHLH and/or anthocyanin structural genes such as *DFR* [30]. Both ppa019522m and ppa010846m are expressed at higher level in red-colored leaf than in green-colored leaf. Therefore, it seems that anthocyanin accumulation in peach leaf is likely coordinatively regulated by both positive and negative regulators of anthocyanin biosynthesis.

The R2R3 MYB TFs are functionally conserved in plants, but may activate distinct sets of structural anthocyanin genes [31]. Structural anthocyanin genes can be divided into two groups, early biosynthetic genes (EBGs, i.e. *CHS*, *CHI*, *F3H*, and *F3′H*) and late biosynthetic genes (LBGs, i.e. *DFR*, *LDOX*, and *UFGT*) [32]. In *Arabidopsis*, *PAP1*, *PAP2*, *MYB113* and *MYB114* control anthocyanin accumulation through regulation of LBGs [33]. Similarly, two MYB genes in grapevine, *VvMYBA1* and *VvMYBA2*, increase anthocyanin biosynthesis in berry through activation of *UFGT* [9]. In contrast, apple *MdMYB10* activates all genes of the anthocyanin biosynthetic pathway, leading to anthocyanin pigmentation in fruit, stem and foliage [12]. In cauliflower, *BoMYB2* specifically activates both regulatory gene *BobHLH1* and structural genes of late anthocyanin pathway, including *BoF3′H*, *BoDFR*, and *BoLDOX* [34].

In this study, the entire set of anthocyanin pathway genes show higher level of expression in red leaves than in green leaves. This indicates that anthocyanin accumulation in peach leaf is regulated at transcriptional level, and *PpMYB10.4*, like the apple *MYB10*, may directly or indirectly activate both EBGs and LBGs. On the other hand, transient color assay reveals that the peach PpMYB10.4, like the apple MdMYB10, interacts with bHLH3 to induce anthocyanin biosynthesis [12]. Previous studies show that the MBW complexes mainly activate LBGs [33,34]. This is also true in our case of the peach transient assay, which shows *PpMYB10.4*, like the grapevine *VvMYBA* genes, increases anthocyanin accumulation in leaves through activation of *UFGT*.

### *PpMYB10.4* represents a novel branch of anthocyanin-activating MYB genes in Rosaceae

Gene duplication has frequently occurred in the evolutionary development of anthocyanin-activating *MYB* genes. For example, multiple clustered *MYB* genes have been reported in grapevine [9] and cauliflower [34]. In this study, two clusters of three anthocyanin regulatory MYB genes in peach have been identified on LGs 3 and 6. The chromosome regions covering these two clusters are not derived from the same ancestral paleochromososme of the eudicot paleoancestor [1]. In apple, two anthocyanin regulatory genes *MYB110a* and *MYB110b* are also clustered in a 60 kb region on chromosome 17 [25], and appear to be related to *MYB10* on the homologous chromosome 9. However, we have not found any clusters of anthocyanin-activating *MYB* genes in the strawberry genome [35]. The genomes of *Fragaria*, *Malus* and *Prunus* are derived by reconstruction of a hypothetical ancestral Rosaceae genome that had nine chromosomes [36]. Thus, it is likely that the clusters of anthocyanin-activating *MYB* genes have evolved after the divergence of peach from other Rosaceae species.

As mentioned above, anthocyanin-activating *MYB* genes in Rosaceae can be divided into two families MYBI and MYBII. Interestingly, the MYBI family is composed of previously reported *MYB* genes that are mainly responsible for anthocyanin accumulation in fruits. For example, the apple *MdMYB110a* contributes to anthocyanin accumulation in fruit cortex late in maturity [25]. Likewise, the peach *PpMYB10.1/2/3* is involved in anthocaynin accumulation in fruit [18,20]. Two alleles of the *MdMYB10* locus *MdMYBA* and *MdMYB1* control red coloration of apple skin although *MdMYB10* is able to induce anthocyanin pigmentation in both fruit (skin and cortex) and foliage due to its constitutive over-expression profile [10,11]. In contrast, the peach *PpMYB10.4* regulates anthocyanin pigmentation in vegetative organs such as leaves, but not in fruit as ‘HYT’ accumulates no anthocyanins in the fruit. The coding sequences of *PpMYB10.4* was aligned the genome sequence databases of apple and *P. mume* using blastn, and two genes MDP0000573302 and Pm001924 from apple and *P. mume*, respectively, are found to have the highest level of similarity to *PpMYB10.4. PpMYB10.4* and its ortholog Pm001924 are diverged from previously reported anthocyanin-activating *MYB* genes in Rosaceae to generate a new gene family MYB II. However, MDP0000573302 is grouped into the MYBI family. Our study shows that MYBI and MYB II genes can be traced to a common ancestor about 70 MYA. The most recent common ancestor of *Malus* and *Prunus* has been dated to 49.42 ± 0.54 MYA [37], and peach has not undergone recent whole-genome duplication [1]. Thus, the two MYB clusters in the peach genome are likely derived from the hypothetical ancestral Rosaceae genome, whereas, the ortholog of *PpMYB10.4* may have been lost in the apple genome after the divergence of apple from peach.

Our study indicates that both *PpMYB10.5* and *PpMYB10.6* are not expressed in peach leaf. Their transcripts are not identified in our previously reported transcriptomes of peach flower and fruit tissues [38]. It has been reported that a siRNA, TAS4-siRNA81(−), targets a set of MYB TFs such as *PAP1* and *MYB113* in *Arabidopsis* [39]. A consensus target sequence (5′-GGCCTCAACCACGAACCTTCT-3′) for TAS4-siRNA81(−) is also found in the third exon of both *PpMYB10.5* and *PpMYB10.6*. This may be responsible for the finding that *PpMYB10.5* and *PpMYB10.6* are not expressed in any tested tissues of peach. In contrast, *PpMYB10.4* contain no target sites for TAS4-siRNA81(−), and its expression is highly induced in red leaves. Several SNPs are found in the promoter region of *PpMYB10.4*. In apple, a SNP 1,661 upstream of the ATG translation start codon of *MYB1* has been reported to co-segregate with red skin color [10]. Thus, it is not yet clear if the activation of *PpMYB10.4* gene could be attributed to single nucleotide mutation in promoter region. In addition, a reciprocal translocation is found between linkage groups 6 and 8 in the F_2_ of an interspecific cross between ‘Garfi’ almond and ‘Nemared’ peach, and the translocation breakpoint is located in the vicinity of the *Gr* locus [40]. This translocation is also found in the F_2_ of a cross between two peach cultivars ‘Akame’ and ‘Juseitou’ [41]. Since ‘Nemared’ and ‘Akame’ are both red-leaved cultivars, it is worthy of further study to ascertain the relationship between this translocation and peach leaf coloration.

### Potential factors affect the change in leaf color of ornamental peach

Peach is a member of a group of temperate deciduous fruit trees, many of which produce green leaves and accumulate anthocyanin during the process of senescence in autumn [2]. The anthocyanin pigmentation provides effective photo-protection during the critical period of foliar nutrient re-absorption. In contrast, the young expanding leaves of ornamental peach ‘HYT’ are red, but the color of leaves fades to green as they mature. This change in leaf color is attributed to decreased expression of *PpMYB10.4*.

Temperature is an important factor that affects anthocyanin biosynthesis in plants [42], which in apple is via expression of *MYB10* [43]. However, *PpMYB10.4* shows no significant difference in expression level between young red leaves grown in different seasons, including spring, summer and autumn (in the high temperatures of Wuhan, China). This is similar to a previous report that the anthocyanin biosynthetic genes have not been strongly down-regulated in grape berry grown at high temperature [44]. Moreover, the anthocyanin contents are also similar between young red leaves grown in different seasons, which is different from the finding that high temperature increases anthocyanin degradation in grape skin [44]. It has been reported that light and hormones play also an important role in anthocyanin biosynthesis [45,46]. Thus, other factors, besides temperature may be responsible for the decreased expression of *PpMYB10.4*. Further studies are needed to clarify what factors play a role in down-regulation of *PpMYB10.4* expression in mature leaves, resulting in the peach leaf color change.

## Conclusions

There are two clusters encoding anthocyanin-activating *MYB* genes in the peach genome, with one gene *PpMYB10.4* in the *Gr* interval on LG 6 being responsible for anthocyanin accumulation in peach leaves. Anthocyanin-activating *MYB* genes in Rosaceae can be divided into two families MYBI and MYBII, which arise from an ancient duplication about 70 MYA. MYBI family is mainly responsible for anthocyanin accumulation in fruits, while MYB II family regulates anthocyanin accumulation in vegetative organs such as leaves.

## Methods

### Plant material

All peach cultivars used in this study are maintained at Wuhan Botanical Garden of the Chinese Academy of Sciences (Wuhan, Hubei province, PRC). A red-leaved cultivar ‘Honyetao’ together with two green-leaved cultivars ‘Baihuabitao’ and ‘Mantianhong’ were selected for quantitative RT-PCR analysis (qRT-PCR) to identify gene responsible for anthocyanin pigmentation in leaves. For cv. ‘Hongyetao’, juvenile and mature leaves were sampled in three different seasons, including spring, summer, and autumn, whereas, the leaves of other cultivars were collected in spring. All samples were immediately frozen in liquid nitrogen, and then stored at −75°C until use.

### Measurement of anthocyanin concentration

Anthocyanin content was assayed following the protocol described by previous study [47]. Briefly, approximately 1 g of tissue was ground to fine powder in liquid nitrogen, and extracted with 5 ml extraction solution (0.05% HCl in methanol) at 4°C for 12 h. After centrifugation at 10,000 g for 20 min, the supernatant was transferred into a clean tube. The sediments were extracted with additional 5 ml extraction solution at 4°C for 6 h. The supernatants were combined and the final volume was measured. Then, 1 ml supernatant was mixed with 4 ml of buffer A (0.4 M KCl, adjusted to pH 1.0 with HCl) or buffer B (1.2 N citric acid, adjusted to pH 4.5 with NaH_2_PO_4_ and NaOH). Absorbance of the mixture was measured at 510 and 700 nm. The anthocyanin content was calculated using the following formula: TA = A * MW * 5 * 100 * V/e, where TA stands for total anthocyanin content as cyanidin-3-O-glucose equivalent (mg/100 g), V for final volume (ml), and A = [(A_510_ - A_700_) at pH1.0] - [(A_510_ - A_700_) at pH 4.5], e is absorbance of cyanindin-3-glucoside (26,900), MW is molecular weight of cyanindin-3-glucoside (449.2). Three measurements for each biological replicate sample were performed.

### RNA-Seq library construction for Illumina sequencing

Total RNA was extracted using the Trizol reagent, followed by RNA cleanup using RNase-free DNaseI (Takara, Dalian, China). PolyA mRNAs were purified using oligo-dT-attached magnetic beads. The purified mRNAs were cleaved into small pieces (200 ~ 500 bp) by super sonication. Cleaved mRNAs were used as templates to construct RNA-Seq library according to our previously reported protocol [38]. The constructed libraries were purified by the AMPure beads, and recovered from the low melting agarose (2%) at the length of about 300 base-pair by the Qiagen Nucleic acid purification kits (Qiagen, CA, USA). Transcriptome sequencing was conducted using Illumina Hiseq2000 sequencer.

### Analysis of gene expression using qRT-PCR

Total RNA was isolated using Universal Plant Total RNA Extraction Kit (BioTeke, Beijing, China) according to the manufacturer’s instructions. The RNA samples were treated with DNase I (Takara, Dalian, China) to remove any contamination of genomic DNA. Three micrograms of total RNA per sample was subjected to cDNA synthesis using Superscript III reverse transcriptase (Invitrogen). A SYBR green-based real-time PCR assay was carried out in a total volume of 25 μL reaction mixture containing 12.5 μL of 2× SYBR Green I Master Mix (Takara, Dalian, China), 0.2 μM of each primer, and 100 ng of template cDNA. Peach gene *PpTEF2* with GenBank accession no. JQ732180 was used as constitutive controls for expression profile analysis of genes. Primer sequences of structural genes related to anthocyanin pathway were the same as reported in our previous study [21]. It is worth noting that four *CHS* genes in peach share high levels of nucleotide sequence identities (94 to 97%) in the coding regions, and their collective expression was investigated using a pair of primers designed from conserved regions. Primer sequences of anthocyanin regulatory genes in peach and anthocyanin structural genes are listed in Additional file 1: Table S1.

Amplifications were performed using Applied Biosystems 7500 Real-Time PCR System. The amplification program consisted of 1 cycle of 95°C for 10 min, followed by 40 cycles of 95°C for 30 sec, and 60°C for 30 sec. The fluorescent product was detected at the second step of each cycle. Melting curve analysis was performed at the end of 40 cycles to ensure proper amplification of target fragments. Fluorescence readings were consecutively collected during the melting process from 60 to 90°C at the heating rate of 0.5°C/sec. All analyses were repeated three times using three biological replicates.

### Estimation of the divergence time of anthocyanin-related MYB genes

The estimation of divergence time of MYB genes was conducted according to our previous reported method [48]. Briefly, the coding DNA sequences were aligned using MUSCLE (multiple sequence comparison by log-expectation) [49] and integrated in MEGA5. The molecular clock was calibrated using two calibration points; divergence of rice-maize (31.0 ± 6.0 MYA) as well as that of monocot-dicot (250.0 ± 40.0 MYA). This calibration served as landmarks to assess the posterior distribution of estimated divergence time points among all samples used. A Bayesian Markov chain Monte Carlo (MCMC) analysis was performed to estimate the divergence dates [50], and the analysis included four independent runs, each with 10 million MCMC steps, and sampled every 1000 generations.

### Dual luciferase assay of transiently transformed tobacco leaves

Two pairs of primers, 5′-CACCATGGATAGTTCTTCGGGAGTGA-3′/5′-GTTATGTTGATAGATTCCAAAGGTC-3′, and 5′-CACCATGGCTGCACCGCCAAGT-3′/5′-CTAGGAATCAGATTGGGGAATTATT-3′ were designed to amplify the coding sequences of *PpMYB10.4* and *PpbHLH3*, respectively, using cDNA templates from young leaves of cv. Hongyetao. The coding sequences were individually inserted into pHEX2 vector under the control of 35S promoter using the LR Clonase II Kit (Invitrogen). A pHEX2 vector containing a *GUS* gene was used as a control for dual luciferase assay. DNA fragment ~ 2.3 kb in size upstream of start codon of *PpMYB10.4* was cloned from cv. Hongyetao using a pair of primers (5′-GGATCTCGCCGCTGTTTCTG-3′/5′-CTCGTACGTCGGATGATGTAACTAGT-3′). The DNA fragment was subsequently inserted into multi cloning site of pGreenII 0800–LUC+ vector, which contains a reporter cassette [51]. Dual luciferase assay was carried out use the same protocol as described by previous study [12].

### Induction of anthocyanins by transient transformation of tobacco

Two-week-old seedlings of *Nicotiana tabacum* grown in greenhouse were used for infiltration. Agrobacterium strain GV3101 was selected for transient color assay. Separate strains containing *PpMYB10.4* or *PpbHLH3* fused to 35S promoter in the pHEX2 vector were infiltrated or co-infiltrated into the abaxial leaf surface. Each infiltration was performed using three leaves for the same plants. The photographs were taken 7 days after infiltration.

### Particle bombardment-mediated transient expression assay in peach leaf

The construct containing *PpMYB10.4* as mentioned above was introduced into young leaf of cv. MTH using the bombardment method as previously reported [52]. Briefly, peach donor material was sterilized young leaves treated by sodium hypochlorite solution. After disinfection, leaves were cut into squares with 1 cm diameter and precultured on MS medium for 24 h. Sub-micron gold particles (0.6 μm) were treated according to the manufacturer’s manual. DNA-coated gold particles are delivered using the PDS-1000/He particle gun (BIO-RAD) according to the manufacturer’s instructions. An empty vector was also transformed as a control. After bombardment, the peach leaves were cultured on MS for 2 days, and cultured media were kept in a growth chamber at 22°C and 50% to 80% relative humidity.

The transformed peach leaves were collected, finely ground in liquid nitrogen, and then subjected to both anthocyanin and RNA extraction. RNA extraction was conducted using the protocol as mentioned above, while anthocyanins were extracted in 1% (v/v) HCl-methanol solution at room temperature and shaken continuously overnight. The extract was centrifuged at 10,000 g for 15 min and the chlorophyll was removed by adding chloroform. The supernatant containing anthocyanins was collected.

## Additional file

Additional file 1: Table S1. qRT-PCR primers of anthocyanin biosynthetic genes in peach and *Arabidopsis*.
