# Supplementary material for: Transcriptome analysis and transient transformation suggest an ancient duplicated MYB transcription factor as a candidate gene for leaf red coloration in peach
Source: BMC Plant Biol. 2014 Dec 31;14:388. doi: 10.1186/s12870-014-0388-y (PMC4302523; doi:10.1186/s12870-014-0388-y)
Supplement: Additional file 1: Table S1. — qRT-PCR primers of anthocyanin biosynthetic genes in peach and Arabidopsis. [file 12870_2014_388_MOESM1_ESM.doc]

**Additional file**

Table S1. qRT-PCR primers of anthocyanin biosynthetic genes in peach and *Arabidopsis*

| Gene | Accession number | Forward primer (5’ to 3’) | Reverse primer (5’ to 3’) | Size of PCR products (bp) |
| --- | --- | --- | --- | --- |
| *PpMYB10.1* | ppa026640m | GAAATGATTGGTGGGAAACC | GTCCTTCTTCTGAAACATTGGT | 156 |
| *PpMYB10.2* | ppa016711m | AAGGCCACAACCAAGAAGA | AACCCAAGACCAGAACCTGT | 147 |
| *PpMYB10.3* | ppa020385m | CACCATCAACAAGGATTGGA | CCGATTGTGGCATATCATCA | 144 |
| *PpMYB10.4* | ppa018744m | AACTGCCAATACTACCCTCATC | TGAAGTGGTCTTCCTCTAGCA | 126 |
| *PpMYB10.5* | ppa022808m | GTCGTTGATTGCTGGAAGAA | TTTATCTTTTTCGGCCTCAGA | 106 |
| *PpMYB10.6* | ppa024617m | GTCGCTGATTGCTGGAAGAC | GAGAAGGTTCGTGGTTGAGG | 159 |
| *PpbHLH3* | ppa002884m | TTGAGACGACAGTTGTCCAGTC | ATTCACATTCTCCTTCACCTTG | 83 |
| *PpbHLH33* | ppa002645m | GTCCTTTGTCTTCAATCCTGG | GTCTGAATAGATGCACTCTTCG | 141 |
| *PpWD40A1* | ppa005733m | TGCAATACGTTTTTGGGACA | GAACCCTTAATCGTGGCAGA | 117 |
| *PpWD40A2* | ppa008187m | TGGCGACTATCTCCGTCTCT | ATTCGTCTGGGTTCGATGTC | 135 |
| *PpTEF2* | TC3544 | GGTGTGACGATGAAGAGTGATG | TGAAGGAGAGGGAAGGTGAAAG | 129 |
